# Social participation and depression among resettled Syrian refugees: examining a moderated mediation of social support and bonding or bridging social capital

**DOI:** 10.3389/fpsyg.2024.1295990

**Published:** 2024-10-28

**Authors:** Charisse M. Johnson-Singh, Mathilde Sengoelge, Karin Engström, Fredrik Saboonchi

**Affiliations:** ^1^Swedish Red Cross University College, Department of Health Science, Huddinge, Sweden; ^2^Karolinska Institute, Department of Global Public Health, Solna, Sweden; ^3^Karolinska Institute, Department of Clinical Neuroscience, Division of Insurance Medicine, Solna, Sweden

**Keywords:** refugees, social networks, social participation, social support, social capital, depression, mental health

## Abstract

**Introduction:**

Aspects of social capital, including social participation and social support, are among the factors influencing refugee mental health after resettlement. However, the mechanisms by which these aspects interact with one another and affect mental health remain unclear. This study investigates whether social participation influences depressive symptoms via social support and whether this influence is more prominent for Syrian refugees participating primarily in coethnic (bonding) networks compared to cross-ethnic (bridging) networks during the early stages of resettlement.

**Methods:**

Using data from a cohort of 464 Syrian refugees recently resettled in Sweden, a moderated mediation analysis was conducted with multigroup structural equation modelling to investigate the mediatory role of social support in the association between social participation and depressive symptoms as well as the moderating role of bonding networks (social participation with other Syrians) versus bridging networks (social participation with Swedes) in this relationship.

**Results:**

Frequent social participation, compared to rare or no participation, was significantly associated with lower depressive symptoms, regardless of whether participation included a broad or limited range of activities. Social support only mediated this relationship for those with primarily bonding networks, indicating that the mediation itself was moderated by network type. While participating in both bridging and bonding networks was associated with lower depressive symptoms, bonding networks amplified the effect of social participation on depressive symptoms via increased social support, resulting in an overall twofold decrease in depressive symptoms compared to those with bridging networks.

**Discussion:**

These findings indicate that the frequency of social participation may be a more important consideration for fostering mental well-being in recently resettled refugees than the specific types of activities. Furthermore, while both bonding and bridging social networks confer mental health benefits, access to coethnic networks in early resettlement appears to provide a particularly crucial source of social support.

## Introduction

Forced displacement due to conflict, persecution, human rights violations, and climate or natural disasters is an ongoing global crisis, and the number of applications for international protection in Europe is approaching the peak levels seen in 2015 (EUAA, 2024). As host countries struggle to face the challenges of integration, a number of international agreements have been adopted to find sustainable solutions to address refugee needs and support host communities, such as the 2018 Global Compact for Refugees (United Nations, 2018). An extensive body of research shows that refugees experience poorer mental health compared to other migrant groups due to a variety of pre- and post-migration factors ranging from torture and violence in their home country and during flight (Sengoelge et al., 2019; Silove et al., 2017; Tinghög et al., 2017) to resettlement problems in host countries related to language difficulties, perceived discrimination, employment restrictions, insecure status, and isolation (Hocking et al., 2015; Solberg et al., 2020). Research has also found that the loss of social networks due to migration (Smyth et al., 2015; Stewart et al., 2010) reduces opportunities for social participation (Lindström, 2005; Niemi et al., 2019) and contributes negatively to refugee psychosocial wellbeing (Bjertrup et al., 2018).

As newly-arrived immigrants begin to settle and integrate into their host country, it is important to develop new social networks (Sluzki, 1992), which provide a means for social participation. Social networks are embedded with “social capital”, an umbrella concept that describes the “features of social organization such as networks, norms, and social trust that facilitate coordination and cooperation for mutual benefit” (Putnam, 1995). Social capital has been conceptualized in various ways, but two main components have been identified: cognitive and structural (Rostila, 2011). Cognitive social capital refers to the perceptions of interpersonal relationships, including the quality of values, norms, beliefs, and solidarity that characterize them (Rostila, 2011; Uphoff, 2000) and has been strongly linked to positive mental health outcomes (Ehsan and De Silva, 2015). Structural social capital refers to the range and frequency of participation in informal and formal social networks that link individuals or groups together (Rostila, 2011). There is limited evidence of any effect of structural social capital on mental health outcomes (Ehsan and De Silva, 2015). A review of literature focusing on social participation, a concept often analogous to structural social capital, shows that, for refugees and asylum-seekers, it is associated with increased psychosocial and subjective wellbeing, as well as decreased psychological distress (Niemi et al., 2019). Social participation is a concept that has been used as an alternative for integration or inclusion (Moore et al., 2001) in order to ascribe a more active role to refugees, and to signify the access of refugees to the host society’s various sub-sectors (Niemi et al., 2019). It is broadly measured in three arenas: formal activities related to societal organizations and structures (e.g., labor market, education, healthcare), community organized groups (sports, religious congregations, political associations), and informal personal networks (Niemi et al., 2019).

While the evidence has established that social participation mitigates poor mental health for refugees and asylum-seekers, little is understood about the underlying mechanisms (Moore et al., 2001). One potential mechanism influencing this relationship is social support, a concept often analogous with cognitive social capital. While the definition of social support lacks consensus (Williams et al., 2004), it has been defined as “the social resources that persons perceive to be available or that are actually provided to them by non-professionals in the context of both formal support groups and informal helping relationships” (Gottlieb and Bergen, 2010). In practice, social support is commonly measured by perceived or received forms of instrumental, emotional, and informational support (Gottlieb and Bergen, 2010) and can have a positive effect on mental health and wellbeing, as well as provide a buffer against potentially stressful events (Cohen and Wills, 1985; Turner and Brown, 2010). A population-based Canadian study found that the association between low social support and mental disorders was strongest among newly-arrived migrants (Puyat, 2013). Perceived social support was found to have a protective effect against depressive symptoms among Eritrean refugees in Ethiopia who were exposed to post-migration living difficulties (Getnet et al., 2019) and low interpersonal support experienced upon arrival by Iraqi refugees in the USA was associated with depressive symptoms 12 months later (LeMaster et al., 2018). While refugees in Europe may be more likely to rely on support from informal social ties, such as via family or friends, rather than from formal, government resources (Hernández-Plaza et al., 2006), there is limited research investigating the ethnic composition of these informal networks and the subsequent differences in social support received from them.

An additional conceptualisation of social capital focuses on differentiating between participation, trust, and cohesion that is built between networks of individuals or groups based on a shared identity, such as ethnicity, known as “bonding” social capital, or whether it links different individuals or groups, called “bridging” social capital (Putnam, 2000; Woolcock and Narayan, 2000). Most studies in the field of migrant mental health focus on the coethnic ties that characterize bonding social capital (De Silva et al., 2005; Tegegne, 2016), though there is mounting evidence that the cross-ethnic ties that characterize bridging social capital are important for psychological adjustment to migrants’ new social environment (Marinucci and Riva, 2021; Repke and Benet-Martínez, 2018; Roblain et al., 2017). Bonding social capital may be particularly important for refugees at the beginning of the resettlement process when they rely on familiar relationships for information and support and has been shown to play a significant role in predicting mental health outcomes (Birman and Tran, 2008; Schweitzer et al., 2006; Teodorescu et al., 2012). During the resettlement phase, bridging social capital becomes more important over time to facilitate social mobility (Zetter et al., 2006), protect from discrimination (Lecerof et al., 2015), and confer a sense of belonging in the host society (Hombrados-Mendieta et al., 2019; Zetter et al., 2006) which in turn, can improve mental health (Teodorescu et al., 2012).

While previous mental health research focused on migrants has identified several aspects of social capital that influence mental health, including social participation (George et al., 2015; Lee and Park, 2018) and social support (Niemi et al., 2019), it is unclear if social participation confers its benefit via social support. Furthermore, it is unknown whether greater social support is conferred by participating in networks of primarily coethnic versus cross-ethnic ties, particularly in the early phases of resettlement. The aim of this study was to investigate the relationship between social participation and depression in a resettled Syrian refugee population, as well as the role of social support and bonding or bridging social networks in this relationship. The following research questions were investigated: (1) Is there a relationship between social participation and depressive symptoms in adult Syrian refugees resettled in Sweden? (2) Is the effect of social participation on depressive symptoms mediated by social support? (3) Are the relationships between social participation, social support and depressive symptoms moderated by type of social network?

## Materials and methods

This cross-sectional study used data from two cohorts of Syrian refugees resettled in Sweden and granted residency on the grounds of asylum. In 2019, Syrians overtook Finns as the largest foreign-born group in Sweden, with nearly 200,000 Syrian-born residents, 64% of which immigrated within the last decade (Tønnessen et al., 2021). The first cohort for this study consisted of individuals from Syria granted permanent residency between 2011 and 2013. In 2016, Statistics Sweden provided a random sample of 4,000 individuals drawn from a sample frame of 9,662 who met these criteria. Of these, 1,215 (30.4%) responded to a baseline questionnaire assessing pre- and post-migration attitudes, experiences and mental health. This response rate equalled 12.6% of the sample frame and was considered adequate representation. Further details of the sampling method and baseline characteristics of this cohort can be found in Tinghög et al. (2017). The second cohort included 129 Syrian asylum-seekers who responded to a similar questionnaire in a study conducted at three asylum-centers in Sweden between March 2016 and May 2018. Additional details regarding data collection and cohort characteristics can be found in Solberg et al. (2020). These 129 respondents were later granted residency and therefore eligible to participate in the present study. The 1,344 eligible respondents from the two cohorts were invited to participate in a follow-up questionnaire, similar to baseline, which included additional questions regarding social participation. The response rate was 34.5%, giving a final study sample of 464 respondents.

### Depressive symptoms

The Hopkins Symptoms Checklist-15 (HSCL-15) is the depression subscale of the HSCL-25 which has been cross-culturally validated (Mollica et al., 1987; Wind et al., 2017). The HSCL-15 consists of 15 questions that assess to what degree specific symptoms have distressed the individual during the last week. Four response alternatives ranging from “not at all” to “very much” were given a score of 1 to 4, respectively. For descriptive purposes, a mean score was calculated for each respondent and those above a threshold of 1.80 are considered to meet the clinical criteria for depression (Oruc et al., 2008). Respondents must have answered at least 13 of the 15 questions to be included in the analysis. The Cronbach’s alpha for this scale was 0.9330 and the McDonald’s omega was 0.9349.

### Components of social capital

Social capital is operationalized in this study with three measures -- social participation, social support, and social network.

#### Social participation

Social participation in this study is based on five items that assess how often, in the past six months, the respondent has participated in a broad range of activities, including: attending or participating in artistic events (such as music, theatre, drawing) or other cultural activities (such as cooking or handicrafts); practicing sports (such as football or swimming) or other games (such as chess, table games or video games); entertainment, leisure or parties; voluntary or humanitarian work in Sweden or in home country (with organizations such as the Red Crescent, the Red Cross, local organizations or non-profit organizations); and met people to express, share or discuss social or political views. Items had the following five response alternatives: once a week or more; approximately twice a month; once a month; less than once a month; and never. Response alternatives were dichotomized as “once a month or more” and “rarely or never.” This cutoff was chosen because a meaningful measure of social participation requires that it occurs with some regularity. The Cronbach’s alpha and McDonald’s omega for the full scale was 0.6936 and 0.6926, respectively, while the dichotomized version had an alpha of 0.6515 and an omega of 0.6461. A cluster analysis was conducted to classify respondents into three social participation groups: respondents who participated in all five types of activities at least once a month or more, titled “*frequent participation in a broad range of activities*”; respondents who participated either in sports/games or entertainment, leisure, or parties at least once a month or more, titled “*frequent participation in a limited range of activities”;* and respondents who rarely or never participated in any of the five types of activities, titled “*rare/no participation*.” The silhouette score for the selected three-cluster solution was 0.4, which is considered “fair,” where “good” has a cut-off of 0.5. Chi-square tests found that the three-cluster solution for social participation was significantly associated with the component questions. The clusters were of sufficiently similar size (within 15% of one another).

#### Social support

The ENRICHD Social Support Inventory (ESSI) was used to measure social support. ESSI consists of seven items that assess structural, instrumental, and emotional support (Gottlieb and Bergen, 2010) and was previously validated in a population of Syrian refugees (Gottvall et al., 2019). Six items are formulated using a 5-point scale ranging from “none of the time” to “all of the time” and ask whether the respondent has someone that: listens when you need to talk; gives good advice about a problem; shows love and affection; helps with daily chores; provides emotional support (talking over problems or helping make a difficult decision); provides as much contact as you would like with someone you feel close to, someone in whom you can trust and confide in? The seventh item is a dichotomous question assessing whether the respondent is currently cohabitating with a partner or not. ESSI is used as a latent variable in the main analysis of this study and cohabitation is used as a formative indicator regressed on the other six (Coltman et al., 2008). For descriptive purposes, a score for overall social support is derived by summing the scores for ESSI items, excluding the items “helps with daily chores” and “cohabitation.” A higher score indicates better social support. The Cronbach’s alpha for these 5 items was 0.9158 and the McDonald’s omega was 0.9177.

#### Social network

Social network was measured by assessing the composition of the respondent’s social networks in relation to all five of the social participation items above with the following question: “How often did you do any of the above activities with people from Sweden?” The five response alternatives were dichotomized into two groups, “once a month or more” and “less than once a month or never,” and were designated as “bridging networks” and “bonding networks,” respectively. While it is not known if those participating in primarily bonding social networks consist mainly of their Syrian coethnics or other migrants, knowledge of the Swedish context points to a considerable proportion being the latter. Over 170,000 Syrian refugees have resettled in Sweden since 2010 (Statistics Sweden, 2020), often in the same neighbourhoods due to both preferences and structural constraints (Malmberg and Clark, 2021).

### Sociodemographic characteristics

Five sociodemographic characteristics were included as potential confounders, all used in categorical form. *Age* was divided into five categories: 20–29 (reference); 30–39; 40–49; 50–59; and 60–67 years-old. *Gender* was defined as man or woman. *Education* was formulated into three categories: completed some or all of primary school (less than 9 years; reference); completed some or all of secondary school (9–12 years); completed some or graduated from post-secondary education (12+ years). *Years of residence in Sweden* was dichotomized as 0–5 years or 6–9. *Cohabitation* was defined as whether the respondent lives with a partner or not. Information for formulating all sociodemographic variables were retrieved from the TPR, except for cohabitation, which came from the questionnaire.

### Statistical analysis

Descriptive statistics of the 464 respondents in the study sample were analysed using SPSS 25.0. Structural Equation Modelling (SEM) was used to investigate the questions of this study, analysed with MPlus using maximum likelihood estimation with robust standard errors (MLR estimator). Social participation was treated as the main exposure of interest, while social support, used as a latent variable, was the mediator, and depressive symptoms the outcome. Social network was included as a potential moderator. Sociodemographic characteristics of age, gender, education, years in Sweden, and cohabitation were included as confounding factors.

#### Moderation models

A multigroup SEM analysis was constructed to investigate the moderating role of social network in the relationships between social participation, support, and depressive symptoms. There were five structural paths representing the individual associations between the exposure, mediator, and outcome; between social participation and depressive symptoms; social support and depressive symptoms; and social participation and social support. *Frequent participation in a broad range of activities* and *frequent participation in a limited range of activities* represent two structural paths to both social support and depressive symptoms (with *rare/no participation* as the reference category).

First, a reference model (Model A) was specified in which all five paths are freely estimated across social network. Next, to determine if any of the five paths are modified by social network, a series of nested models were fit, beginning with fixing equality constraints to all five pathways, and then sequentially releasing them. The Satorra-Bentler scaling correction likelihood ratio (S-BΔχ2) (Hu and Bentler, 1999; Kline, 2005; Satorra and Bentler, 2010) was used to compare each model to the reference and for selecting the best fitting model. Other fit indices including chi-square (χ2), root mean square error of approximation (RMSEA) with 90% confidence interval (CI), comparative fit index (CFI), standardized root mean square residual (SRMR), were presented to provide an overview of the fit of each individual model. Moderation was indicated if the selected model contained structural paths that vary across social network.

#### Mediating function of social support

The mediating role of social support was investigated after selecting the best fitting multigroup SEM model. The direct effect of social participation on depressive symptoms and the indirect effect via social support was estimated using Maximum Likelihood estimator with 95% bias corrected bootstrap (5,000 resampling) CI.

#### Moderated mediation

Whether social network moderates the mediating role of social support was estimated using the Wald test (Ryu and Cheong, 2017) to determine the group difference in the indirect effects for bonding and bridging social networks.

### Ethnics approval

The study was approved by the Stockholm Regional Ethical Review Board (number: 2015/1463-1431 and 2016/549-32).

## Results

Table 1 shows the descriptive characteristics of the study sample. The mean age of the respondents was 43 years-old, the majority were men (65%), and just over 70% were living with a partner. Nearly half of the respondents had at least some post-secondary education and 70% had been living in Sweden five years or less. Half of the respondents reported good social support and 39% had *frequent participation in a broad range of activities*, 38% had *frequent participation in a limited range of activities*, and 23% reported *rare/no participation*. Depressive symptoms at or above the clinical cutoff (1.80) was reported in 42% of respondents.

**Table 1:** Descriptive statistics of sociodemographic characteristics, social capital indicators, and depressive symptoms (*N* = 464).

	*n*	%
Gender		
Men	302	65.1
Women	162	34.9
Age group, in years		
Mean (SD)	464	43.1 (11.3)
20–29	53	11.4
30–39	131	28.2
40–49	139	30.0
50–59	105	22.6
50–67	36	7.8
Cohabitancy with partner		
Cohabitating	325	71.3
Not cohabitating	131	28.7
Level of education		
9 years or less	141	30.8
9–12 years	109	23.8
> 12 years	208	45.4
Years in Sweden		
1–5 years	327	70.6
6–9 years	136	29.4
Social network		
Bonding	294	67.6
Bridging	141	32.4
Social support		
Mean (SD)	440	17.1 (5.8)
High	216	49.1
Low	224	50.9
Social participation		
Frequent participation in broad range of activities	164	38.9
Frequent participation in limited range of activities	159	37.8
Rare/no participation	98	23.3
Depressive symptoms*		
Mean (SD)	444	1.7 (0.6)
> 1.80	187	42.1
≤ 1.80	257	57.9

*Participants with a mean depressive symptom score above 1.80 are considered to meet the clinical criteria for depression.

### Social network moderation in the association between social participation, social support, and depressive symptoms

**Figure 1:**
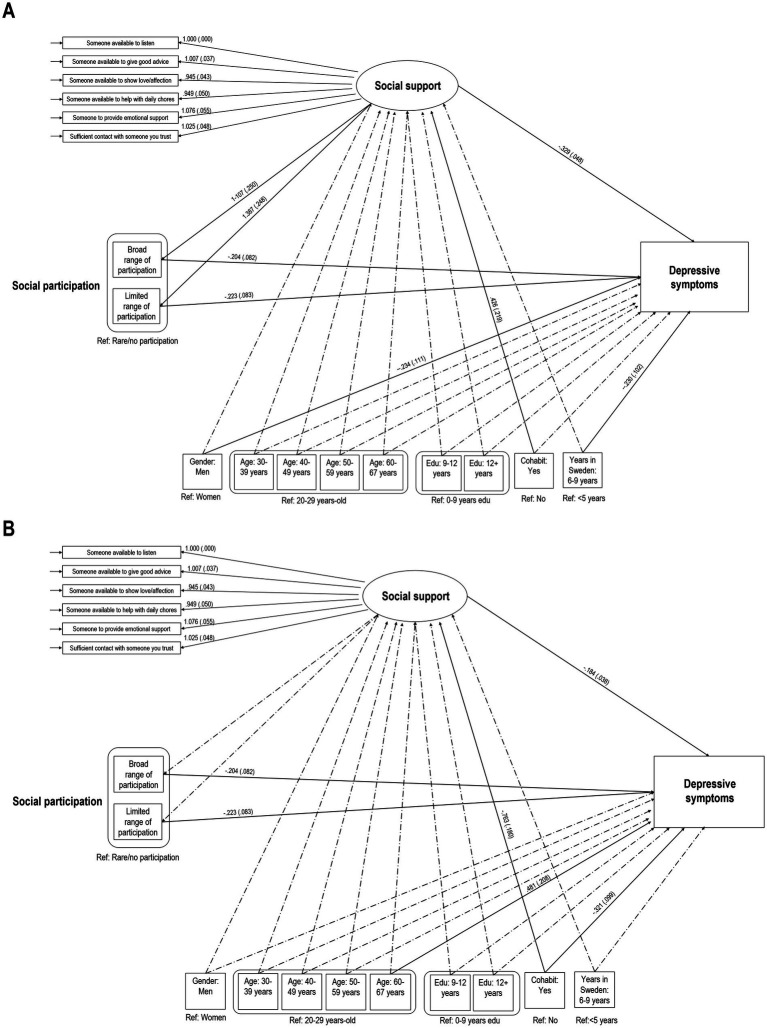
Bonding social network **(A)** and bridging social network **(B)**. Full structural equation model of depressive symptoms on the latent variable social support. The displayed estimates for regression weights are unstandardized (beta) with standard error in parentheses. Significant weights are indicated by solid-line arrows and dashed-line arrows indicate nonsignificant associations. Values for residual variances of indicator variables (boxes) are not included in the figure for readability issues (empty arrows signify that variances were part of the full structural equation model).

To determine whether social network moderates the structural model, equality constraints across social network were imposed on various paths between social participation, social support, and depressive symptoms, controlled for age, gender, education, years in Sweden, and cohabitation. Table 2 shows the fit indices for each model and the ^S-B^Δχ2 tests for comparing with the reference, in which the structural weights for all five paths were allowed to vary (Model A). All models, except two, showed significantly worse fit compared to the reference. The two that did not have significantly worse fit were: Model C, which imposed equality constraints across the two social network groups on the association between social participation and depression as well as the associations between social participation and social support; and Model F, with equality constraints between social participation and depression. Comparing these two models with ^S-B^Δχ2 showed that Model C had a significantly worse fit compared to Model F. Model F was, consequently, selected as the best fitting model, indicating that social network moderates the associations between social participation and social support as well as between social support and depressive symptoms, but not between social participation and depressive symptoms. The resulting structural model is shown in Figure 1 for both bonding networks (panel a) and bridging networks (panel b).

**Table 2:** Fit indices of measurement models. The selected model is in bold.

Model	χ2	df	*p*	CFI	RMSEA (90% CI)	SRMR	Δ S-Bχ2	∆df	*p*
Model A: Reference (freely estimated)	183.365	146	0.0196	0.979	0.036 (0.015–0.052)	0.043			
*Test fit of models with various equality constraints against reference model (A)*									
Model B: SP to Dep + SP to SS + SS to Dep	196.061	151	0.008	0.974	0.039 (0.021–0.054)	0.051			
Model B vs. A							13.956	5	0.016
Model C: SP to Dep + SP to SS	183.365	150	0.015	0.977	0.037 (0.017–0.052)	0.045			
Model C vs. A							7.193	4	0.126
Model D: SP to Dep + SS to Dep	191.277	149	0.0111	0.976	0.038 (0.019–0.053)	0.049			
Model D vs. A							7.862	3	0.049
Model E: SP to SS + SS to Dep	193.854	149	0.0079	0.974	0.039 (0.021–0.054)	0.05			
Model E vs. A							12.326	3	0.006
**Model F: SP to Dep**	**185.293**	**148**	**0.0204**	**0.979**	**0.036 (0.015–0.051)**	**0.043**			
Model F vs. A							1.872	2	0.392
Model G: SP to SS	188.106	148	0.0144	0.977	0.037 (0.018–0.052)	0.044			
Model G vs. A							6.339	2	0.042
Model H: SS to Dep	189.147	147	0.0109	0.976	0.038 (0.019–0.053)	0.048			
Model H vs. A							5.062	1	0.024
*Test fit of non-significant models*									
Model C vs. F							6.343	2	0.042

SS, social support; SP, social participation; Dep, depression. CFI Comparative Fit Index; RMSEA Root Mean Squared Error of Approximation; CI Confidence Intervals; SRMR Standardized Root Mean Square Residual; Δ S-Bχ2 Satorra-Bentler scaled Chi Square difference; ∆df difference degrees of freedom.

### Individual associations between social participation, social support, and depressive symptoms

There was no difference in the association between social participation and depressive symptoms between the two social network groups. For those with bonding networks, respondents that reported either *frequent participation in a broad range* or a *limited range of activities* had significantly better social support and significantly fewer depressive symptoms compared to those with *rare/no participation*. For those with bridging networks, respondents that reported either *frequent participation in a broad range* or *limited range of activities* show significantly fewer depressive symptoms compared to those with *rare/no participation*. While there is no significant association between social participation and social support, higher social support is significantly associated with fewer depressive symptoms.

### Social network moderation of social support mediation

Table 3 shows the results of the mediation analysis, decomposed into the total and direct effect of social participation on depressive symptoms and the indirect effect via social support. The pattern of mediation is significantly different between bonding and bridging network groups, for both the effect of *frequent participation in a broad* (Wald = 12.044, df = 1, *p* < 0.001) and *limited range of activities* (Wald = 15.888, df = 1, *p* < 0.001) on depressive symptoms, indicating that social network moderates the mediation. For those with bonding networks, the direct and total effects of social participation on depressive symptoms are significant, as is the indirect effect of social support, indicating partial mediation. Conversely, those with bridging networks show significant direct and total effects, while the indirect effect of social support does not, indicating that there is no evidence of mediation for this group.

**Table 3:** Estimates (Est.) and 95% confidence intervals (CI) for the total, direct, and indirect effects of social support in mediating the relationship between social participation and depressive symptoms, stratified by social network. Associations that are statistically significant are in bold.

Social participation	Bonding social network		Bridging social network	
	Est.	CI	Est.	CI
*Broad range of participation* vs. *rare/no*				
Direct	**−0.204**	**[−0.374, −0.041]**	**−0.204**	**[−0.374, −0.041]**
Indirect (via social support)	**−0.365**	**[−0.589, −0.134]**	−0.041	[−0.124, 0.025]
Total	**−0.569**	**[−0.849, −0.315]**	**−0.245**	**[−0.428, −0.069]**
*Limited range of participation* vs. *rare/no*				
Direct	**−0.223**	**[−0.395, −0.060]**	**−0.223**	**[−0.395, −0.060]**
Indirect (via social support)	**−0.457**	**[−0.716, −0.231]**	−0.052	[−0.130, 0.006]
Total	**−0.680**	**[−0.960, −0.429]**	**−0.274**	**[−0.454, −0.104]**

Associations that are statistically significant are in bold.

## Discussion

The purpose of this study was to investigate the pattern of associations between depressive symptoms and three important aspects of social capital, namely social participation, social support, and social network, in a group of Syrian refugees resettled in Sweden. Given the known interrelationship between social support and depression (Cohen and Wills, 1985; Turner and Brown, 2010), a specific aim was to examine whether the potential influence of social participation on depression would operate via enhanced social support, i.e., whether social support mediates the association between social participation and depression. Furthermore, the role of social network was explored as a potential moderator of this relationship, given that having coethnic connections as well as those from the host country have been identified as potentially important integration factors for newly-arrived immigrants (Lewis, 2020; Zetter et al., 2006) that may also influence mental health (De Silva et al., 2005; Ehsan and De Silva, 2015).

The best fitting model to the empirical data in the present study demonstrated that frequent social participation, irrespective of whether it was in a broad or limited range of activities, was significantly associated with lower depressive symptoms for both those with bonding and those with bridging networks. No difference was found between the social network groups for the direct effect of social participation on depressive symptoms. However, the indirect effect showed that social support partially mediated this association for those with bonding networks, demonstrating that social participation was significantly associated with higher social support, which in turn was significantly associated with two-fold lower depressive symptoms compared to those with bridging networks. Applying Cohen & Wills’ main-effect and stress-buffering models to these findings, the higher social support experienced by those participating frequently in bonding networks could have a positive impact on mental health directly, or offer protection against the negative effects of stressful events or circumstances (Cohen and Wills, 1985). These results partially align with previous research that has found that the strong, close ties that characterize bonding networks can have either a positive or negative influence on mental health (De Silva et al., 2005). Bonding networks may place demands and social pressures on individuals as well as reify group norms that adversely impact mental wellbeing (Mitchell and LaGory, 2002; Rostila, 2010). At the same time, close connections with coethnics facilitate a sense of trust, social stability, social inclusion, and combat isolation (De Silva et al., 2005; Kawachi and Berkman, 2000). Furthermore, accessing bonding without bridging networks was found to be detrimental to mental health (Stafford et al., 2008). This is not supported by the findings of this study, given that respondents with bonding networks had few bridging ties. It is possible that the social participation variables in this study do not include all activities whereby respondents may interact with Swedes, thereby not capturing all possible bridging connections and underestimating their impact on depressive symptoms. Alternatively, it is possible that the resettlement context of Sweden, which allows refugees to access to various forms of practical support in the initial phases such as housing and allowance (Ahlén and Palme, 2020; Migrationsverket, 2020), may lessen the burden that otherwise might be placed on individuals to support one another.

The significant relationship between social participation and depressive symptoms was only partially mediated by social support for respondents with bonding networks and not at all for those with bridging networks. This indicates that social participation also operates via other mechanisms than social support. Given that social participation implies a degree of embeddedness within a social network, individuals may be influenced by group norms and behaviours that promote mental wellbeing, as well as facilitate a sense of purpose, belonging, security and self-worth which can have a positive effect on mental health (Kawachi and Berkman, 2001). Furthermore, being integrated into a social network would also likely increase access to information regarding health services and encourage help-seeking (Gourash, 1978; Kawachi and Berkman, 2001; Kim et al., 2015). Another potential mechanism is physical activity, given that participating in sports is a form of social participation included in this study that many newly-arrived immigrants engage in (Xin, 2018) and has been shown to protect against and even contribute to remedying depression (Helgadottir, 2016).

The findings of this study echo previous evidence that various aspects of social capital can have beneficial effects on mental health outcomes in resettled refugees. Niemi et al. (2019) conceptualized refugee and asylum-seeker social participation as a framework of opportunities and actors, rather than simply an action to be taken. The framework is delineated into two components: the host society structure –consisting of the regulatory policies and established societal and community organizations that dictate participation accessibility— and individual agency. It is important for policy-makers to identify the facilitators as well as potential barriers to social participation and for developing both bonding and bridging ties. From a structural stand-point, a 2018 systematic review by Huaibo Xin identified a number of settings or tools whereby new immigrants built bonding and/or bridging social networks, including: networks of family and friends; settings of religious practice or recreation; schools; formal ethnic associations; informal community groups; and via social media or other technology.

There are several factors that policy-makers should consider in creating a resettlement context that facilitates individual agency for building social capital, given that social capital development can have a long-term impact on future health outcomes (Tegegne, 2016). First, identifying and treating mental illness as soon as possible in the resettlement process is important, as depression has been linked to lower engagement with one’s own ethnic community (Nickerson et al., 2019). Second, lack of trust in the host society is also a barrier to developing bridging ties (Nickerson et al., 2019). Third is understanding the relative importance of various social resources at different stages of the resettlement process. It is theorized that bonding ties are particularly important in the beginning of the resettlement process and that bridging ties become more important over time (Zetter et al., 2006). Sweden’s Syrian refugee population represented in this study reported that over 75% frequently participated in social activities, while nearly 70% and just under 30% had bonding or bridging networks, respectively. While bonding networks can have a stronger main effect on mental health outcomes given the nature of the strong, close ties that characterize it (De Silva et al., 2005; Ehsan and De Silva, 2015), the fact that 70% of respondents had been in Sweden less than 5 years may also explain why the influence of bonding networks compared to bridging networks is particularly apparent in this study. A follow-up of these respondents may reveal that the importance of bridging networks with regards to mental health increases with time.

### Strengths and limitations

The present study assessed the social participation of Syrian refugees for a broad range of activities as well as whether those activities included frequent interaction with Swedes. Social participation in the form of bridging networks is a construct that is rarely measured empirically in migration health research, and when it is, only general participation in a broad range of activities is often used as a proxy for regular interaction between individuals with different ethnicities (Daoud et al., 2016; Johnson et al., 2017). Another strength of this study is the use of validated measures for social support and depression. The ENRICHD scale used to assess social support was previously validated in a Syrian refugee population and demonstrated good psychometric properties (Gottvall et al., 2019). Likewise, the HSCL-25 depression subscale has been cross-culturally validated, including within refugee populations (Tinghög and Carstensen, 2010).

The study population at follow-up was used because social participation, the main exposure of interest, was not measured at baseline. As a result, selection bias may have been introduced at two points—first, due to the non-response during data collection at baseline and second, due to the dropout of respondents between baseline and follow-up. This could limit generalizability with regards to the extent to which the study sample reflects the general population of Syrian refugees in Sweden. Furthermore, these results may not be generalizable to other ethnic groups, as there is some evidence that different ethnic groups value different forms of social support and may vary in its effect on mental health (du Plooy et al., 2019). Selection bias may also be present for the social participation and social support variables, as those with more social resources may likely have greater trust in institutions and thereby may be more likely to fill-out the questionnaire. While this could result in prevalence estimates that reflect higher social participation and social support as well as fewer depressive symptoms, it is unlikely to affect the associations between these variables (Rothman and Greenland, 2005).

While the advanced modelling methods for assessing mediation and moderated mediation allowed for simultaneously estimating the associations of various structural paths, implications regarding causality should be interpreted with caution due to the cross-sectional design of the study. Some reverse causality, in which mental health affects participation, may be reflected in this study’s results. Previous research in refugee mental health shows mixed results in this regard, depending upon the network composition. Nickerson (2019) found that while psychological symptoms in Vietnamese refugees resettled in Australia did not impact engagement with other ethnic communities, they did decrease the interaction with others from Vietnam. This indicates that the association between social participation and depressive symptoms may be more reliable for respondents with bridging versus those with bonding networks. Due to power considerations, respondents with depression (i.e., depressive symptoms above the clinical threshold of 1.80) at baseline were not excluded from the study sample, as could be done to attempt to address potential reverse causality. Instead, a sensitivity analysis was conducted on a subset of the data— those among the baseline refugee cohort who reported depression at follow-up— which found that there was no significant difference in social participation based on mental health at baseline for those who reported depression at follow-up. This indicates that the potential for reverse causality between the exposure and outcome, while still potentially present, would not greatly alter the results. Nevertheless, future longitudinal studies are needed to control for directionality.

Additionally, while the multigroup analysis found that there was no difference between social network groups with regards to the direct effect of social participation on depressive symptoms, this association may be underpowered to detect differences due to the small cell count of rare/no participation among the bridging network group. Finally, although this study accounted for several potential confounding factors (age, gender, education, and cohabitation status), there may have been residual confounding due to excluding other potentially important factors, particularly those relevant to pre-migration circumstances.

## Conclusion

This study explored how aspects of social capital are connected in their effect on mental health. The frequency of social participation may be more important than the specific type of social activities in which refugees participate with regards to reducing depressive symptoms. Furthermore, while social participation can be viewed as beneficial for the mental health of refugees in general, it particularly enhances the effect of social support for those in the beginning stages of resettlement who have access to the coethnic networks that characterize bonding social capital. These results have implications for policy-makers in considering how to facilitate opportunities for social participation, both among coethnic communities and in the host society, as a means to ensure the mental health of resettled refugees.

## Data Availability

The statistical code is available from the corresponding author. Under Swedish law and ethical approval, individual-level data of this kind cannot be publicly available. Individual-level data can be made available on reasonable request as long as it is in line with Swedish law and ethical approvals.

## Author’s note

A previous version of this paper was published as part of a doctoral dissertation (Johnson-Singh, 2021).
